# Down-regulation of Cav1.3 in auditory pathway promotes age-related hearing loss by enhancing calcium-mediated oxidative stress in male mice

**DOI:** 10.18632/aging.102203

**Published:** 2019-08-19

**Authors:** Fan Qi, Rongsheng Zhang, Jin Chen, Fei Zhao, Yanbo Sun, Zhihui Du, Dan Bing, Pengjun Li, Shengli Shao, Hongmei Zhu, Hanqi Chu

**Affiliations:** 1 Department of Otolaryngology-Head and Neck Surgery, Tongji Hospital, Tongji Medical College, Huazhong University of Science and Technology, Wuhan, Hubei 430030, China; 2 Cancer Research Institute, Tongji Hospital, Tongji Medical College, Huazhong University of Science and Technology, Wuhan, Hubei 430030, China; 3 Jinzhou Medical University, Liaoning, Jinzhou 121000, China

**Keywords:** CaV1.3, ARHL, ROS, intracellular calcium, apoptosis

## Abstract

In this study, age related Cav1.3 expression in cochlea and auditory cortex of C57BL/6J male mice was evaluated. It was found that the expression of Cav1.3 in cochlea decreased with aging whereas this phenomenon was not observed in neuron of auditory cortex. The correlation between decreased expression of Cav1.3 and age-related hearing losses was studied in vitro, after Cav1.3 was knocked out, the rate of apoptosis of hair cells increased after being subjected to ROS stresses, accompanied with enhanced senescence. Further, Cav1.3 knock down also interfered with the electrophysiology of hair cells. The effect was further confirmed in vivo, after Cav1.3 knocked down by injection of AAV, hearing impairment was observed in C57BL/6J male mice subjected to senescence and this was accompanied by increased loss of hair cells in cochlea. The effect was further confirmed in 3D organ culture, increased loss of hair cells after Cav1.3 was knocked down under ROS stresses.

Mechanistically, Cav1.3 knock out resulted in decreased intracellular calcium which subsequently reduced the inactivation of ROS from complex I, and finally resulted in increased intracellular ROS and enhanced senescence.

Collectively, these findings confirmed that Cav1.3 could protect cells in auditory pathway from oxidative stresses, and decreased expression of Cav1.3 in auditory pathway could contribute to hearing losses by enhancement of calcium-mediated oxidative stress.

## INTRODUCTION

Presbycusis or age-related hearing losses (ARHL) is the most common cause of hearing loss [1, 2]. Overall, 10% of population experience hearing loss which is sufficient to influence communication, and the rate increases to 40% in the population older than 65 years [3, 4]. The high prevalence of presbycusis causes severe social and health problems [5]. It has been found that oxygen free radical, glutamate toxicity, and Ca^2+^ overload are closely related to ARHL [6–8]. However, the underlying mechanism for ARHL are still obscure.

Cav1.3 is a voltage-gated calcium channel which plays an important role in diverse cell functions. It is mainly expressed in CNS, mediated persistent Ca^2+^ influx, sustain plateau potential, support peacemaking [9], and has a role in hair cell development [10–12]. Moreover, it has been found that Cav1.3 calcium channel in the stria vascularis contribute to the generation and maintenance of the end cochlear potential. Chen et al [13] reported that the expression of Cav1.3 in cochlea of C57BL/6J was gradually decreased when the mice got older accompanied by increased hearing threshold, which indicated a possible association between decreased expression of Cav1.3 and ARHL, but the underling mechanism is still unknown.

It is a common understanding that oxidative stress and mitochondrial dysfunction play a major part in aging [14–18], ARHL can also be caused by ROS and mitochondrial dysfunction [19], Menardo et al [20] reported that oxidative stress, altered level of antioxidant enzymes, and decreased activity of complex I, II, and IV could trigger apoptotic cell death pathways. Ca^2+^ primary promotes ATP synthesis by stimulating enzymes of Krebs cycle and oxidative phosphorylation in the mitochondria. However, it can also diminish ROS from both complexes I and III [21], therefore, changes of intracellular Ca^2+^ could influence ROS generation and subsequently causes ARHL.

In this study, age related Cav1.3 expression in cochlea and auditory cortex of C57BL/6J male mice was evaluated and it was confirmed that Cav1.3 expression in cochlea was decreased with aging whereas this phenomenon was not observed in neuron of auditory cortex. The effect of Cav1.3 knock down or knock out on hair cells when facing aging induction or ROS stresses was observed in vitro and further confirmed in vivo, the underling mechanism was also investigated.

## RESULTS

### Age-related expression of Cav1.3 in cochlea

**Figure 1:**
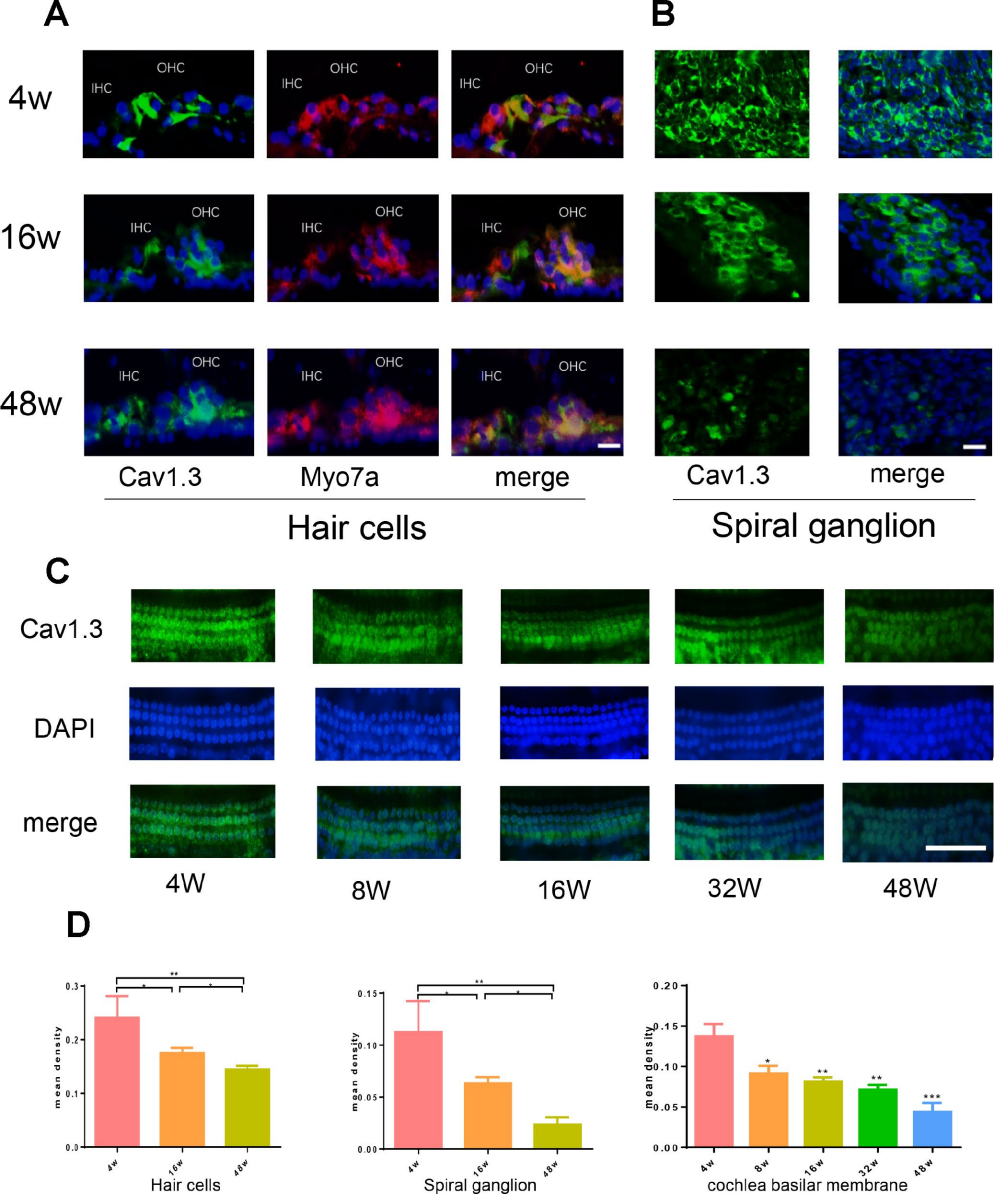
**Age-related Cav1.3 expression in cochlea.** (**A**, **B**) immunofluorescence of CaV1.3(green) and Myo7a (red) in the organ of Corti (left) and spiral ganglion (right) (magnification, ×400), nuclei was visualized by DAPI (blue). (**C**) the immunofluorescent staining for CaV1.3 (green) in the whole cochlear basilar membrane. (**D**) quantitative analysis of CaV1.3 expression in hair cells, spiral ganglion and cochlea basilar membrane.

The expression of Ca_V_1.3 calcium channels in cochlea of C57BL/6J male mice was detected by immunofluorescence. As shown in Figure 1A, 1B, Intense-labeling for CaV1.3 is visible in the organ of Corti (OC) (Figure 1A) and spiral ganglion (SG) neurons (Figure 1B). The labeling was significantly reduced both in OC and SG neurons of the aging mice, only very weak labeling for CaV1.3 was visible. The immunofluorescence staining for CaV1.3 in the OC segment explants (Figure 1C) showed that the expression of CaV1.3 in hair cells gradually decreased with age.

### Age-related expression of Cav1.3 in auditory pathway

**Figure 2:**
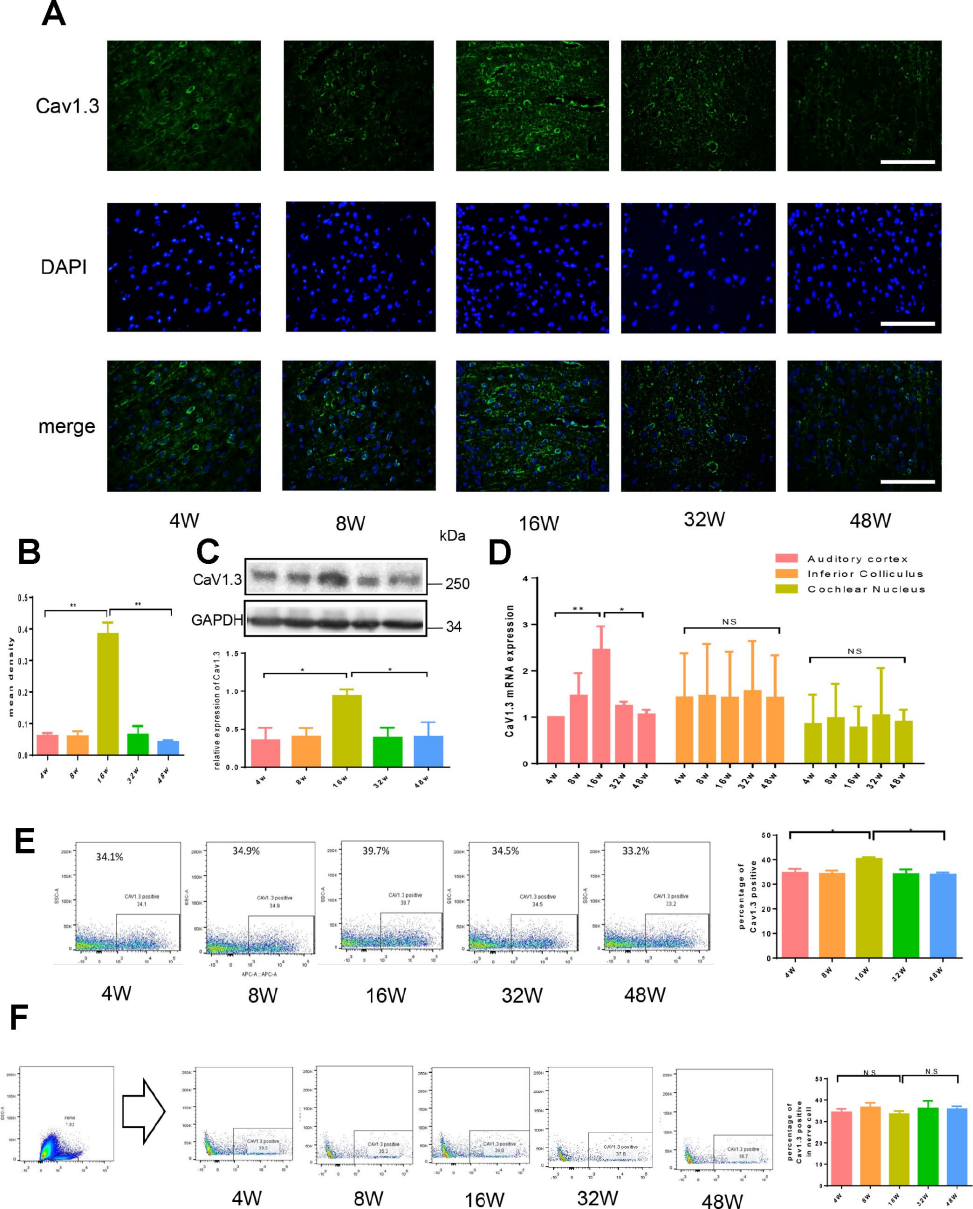
**Age-related expression of Cav1.3 in auditory pathway.** (**A**) The immunofluorescence of CaV1.3 in the auditory cortex (green, magnification, ×400). (**B**) the quantitative analysis of CaV1.3 expression in the auditory cortex. (**C**) the western-blotting analysis of CaV1.3 expression in auditory cortex (top), the bottom panel is the quantitative analysis. (**D**) the mRNA expression of CaV1.3 in auditory cortex, inferior colliculus and cochlear nucleus. (**E**) CaV1.3 expression in auditory cortex was analyzed by flow cytometry, the right panel is the quantitative analysis. (**F**) Cav1.3 expression in neurons of auditory cortex, the neuron cells were gated as NeuN+, the right panel is the quantitative analysis. Error bars represent mean ± s.d.; *P<0.05; **P < 0.01; ***P < 0.001; n.s. not significant; by one-way analysis of variance (ANOVA) (**B**–**F**).

The presence Cav1.3 protein in auditory cortex was also explored in this study. The results showed that, some but not all neurons in auditory cortex of of C57BL/6J male mice expressed Cav1.3 at moderate level, as shown in Figure 2A. Age-related expression of Cav1.3 was observed by immunofluorescence which indicated that its expression in auditory cortex increased before 16 weeks and decreased after 16 weeks (Figure 2A, 2B). The changes in expression of Cav1.3 was further confirmed by real-time PCR, flow cytometry and western-blot (Figure 2C–2E). To further confirm Cav1.3 expression specifically in neurons of auditory cortex, the neuron cells were gated as NeuN+. As shown in Figure 2F, Cav1.3 expressed in auditory cortex-derived neurons remained constant between different age groups. The mRNA expression of Cav1.3 in inferior colliculus and cochlear nucleus were also evaluated, as shown in Figure 2D. No significantly difference in Cav1.3 expression was observed in cochlear nucleus and inferior colliculus.

### Hair cells were vulnerable to ROS injury after Cav1.3 was knocked out

**Figure 3:**
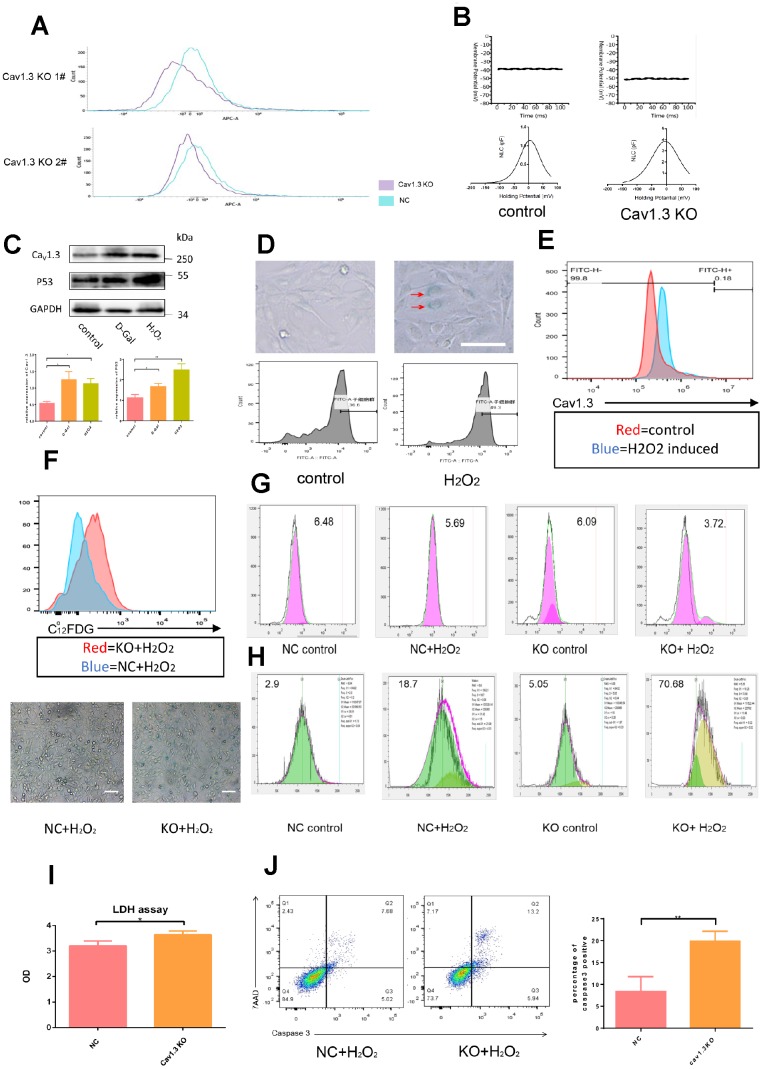
**Hair cells were vulnerable to ROS injury after Cav1.3 was knocked out.** (**A**) the effect of CaV1.3 knock out in HEI-OC1 was analyzed by flow cytometry. (**B**) membrane potential (top) and non-linear capacitance (NLC) (bottom) studies in WT HEI-OC1 and CaV1.3 KO HEI-OC1 cells (n=5). (**C**) western-blotting analysis of CaV1.3 and p53 expression in control and senescence HEI-OC1 cells induced by D-galactose (D-Gal) or hydrogen peroxide (H_2_O_2_), the bottom panel is the quantitative analysis. (**D**) β-Galactosidase staining (top) and C12FDG staining (bottom) of control and senescent HEI-OC1 cells induced by H_2_O_2_. (**E**) flow cytometry analysis of CaV1.3 in control and H_2_O_2_ induced HEI-OC1 cells. (**F**) C12FDG staining (top) and β-Galactosidase staining (bottom) of NC (negative control) and KO (CaV1.3 knock out) HEI-OC1 cells after H_2_O_2_ induction. (**G, H**) CFSE staining and red dot staining of NC and KO HEI-OC1 cells with or without H_2_O_2_ induction. (**I**) LDH assay of NC and KO HEI-OC1 cells after H_2_O_2_ induction (n=3). (**J**) caspase-3/7-AAD staining of NC and KO HEI-OC1 cells after H_2_O_2_ induction (n=3). Error bars represent mean ± s.d.; *P<0.05; **P < 0.01; ***P < 0.001; n.s. not significant; by one-way analysis of variance (ANOVA) (**I**, **J**).

To investigate the underlying role played by Cav1.3 in electrophysiology, Cav1.3 was knocked out by crisper technology in hair cell line HEIOC1, and the effect of knock out was confirmed by flow cytometry (Figure 3A). As shown in Figure 3B, wild type HEIOC1 had membrane potential of about -40mV, Cav1.3 knock out decreased membrane potential to about -50mV. Non-linear capacitance is considered as the electrical signature of prestin motor function, prestin NLC is bell-shaped and voltage-dependent. NLC traces using the parameters Qmax (maximum nonlinear charge transfer), Vmax (voltage at peak capacitance), and Clin (linear capacitance). NLCmax= (Cmax- Clin) is the peak value of NLC. As shown in Figure 3B, mean NLCmax of wild type HEIOC1 were 0.7pF, Cav1.3 knock out enhanced the mean NLCmax to 5.5pF which indicated that Cav1.3 knock out enhanced motor function of prestin, possibly due to progressive translocation of prestin from the cytoplasm to the plasma membrane.

To investigate the relationship between Cav1.3 and age-related hearing losses, hair cell line HEI-OC1 was induced by hydrogen peroxide and D-galactose to produce senescence. The effect of induction was evaluated by Western-blotting based P53 detection (Figure 3C), and the effect of induction was confirmed by C12FDG stain and β-Galactosidase stain (Figure 3D). Enhanced expression of Cav1.3 was observed in senescent cells (Figure 3C), which was further confirmed by flow cytometry (Figure 3E), implying a possible oxidative stress-protection role played by Cav1.3. To confirm this, the viability of the cells subjected to oxidative stress by administration of hydrogen peroxide was observed, Cav1.3 knock out aggravated senescence of hair cells accompanied by enhanced proliferation arrest, and increased S phase (Figure 3F-H). After induction of apoptosis by hydrogen peroxide, the released LDH in supernatant was increased significantly after Cav1.3 was knocked out (Figure 3I), this was further confirmed by caspase-3/7-AAD stain (Figure 3J). Interestingly, apoptosis of HEI-OC1 could not be detected by traditional Annexin V stain (including positive control of apoptosis) due to unknown reason (data not shown).

### Cav1.3 knock out decrease intra cellular calcium and subsequently result in reduction of complex I derived ROS inactivation

**Figure 4:**
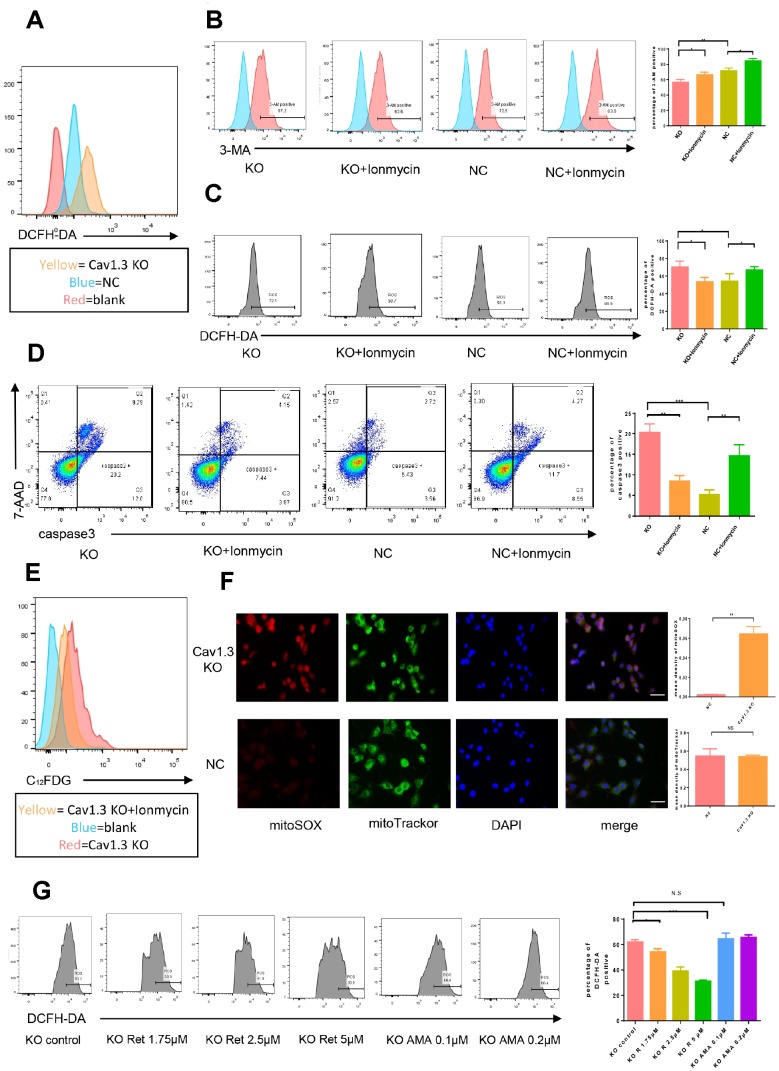
**Cav1.3 knock out decrease intra cellular calcium and subsequently result in reduction of complex I derived ROS inactivation.** (**A**) intra-cellular ROS detection by flow cytometry (n=3). (**B**–**E**) the intra cellular calcium, intra cellular ROS, caspase-3/7-AAD staining and C12FDG staining of NC and KO HEI-OC1 cells with or without Ionmycin (n=3). (**F**) immunofluorescence of mitoSOX (red) and mitotrackor (green) in NC and KO HEI-OC1 cells, nuclei was visualized by DAPI (magnification, ×400, scal bar: 50μm), the right panels are the quantitative analysis of mitoSOX (top) and mitoTrackor (bottom). (**G**) the intra cellular ROS of KO HEI-OC1 cells with or without gradient Retenone (Ret) and Antimycin A (AMA)(n=3), the right panel is the quantitative analysis. Error bars represent mean ± s.d.; *P<0.05; **P < 0.01; ***P < 0.001; n.s. not significant; by one-way analysis of variance (ANOVA) (**B**, **C**, **D**, **G**).

The underlying mechanism responsible for the vulnerability of hair cells to ROS after Cav1.3 was knocked out was investigated. It was found that Cav1.3 knock out induced significant up-regulation of intra-cellular ROS (Figure 4A). As expected, the intra cellular calcium was decreased as Cav1.3 was KO (Figure 4B). After administration of Ionmycin, the intra cellular calcium level was partly rescued, accompanied with decreased intra cellular ROS (Figure 4B–C). ROS-mediated apoptosis and senescence was also partly alleviated (Figure 4D-E), which implied that Cav1.3 knock out induced up-regulation of intra cellular ROS was partly induced by decreasing of intra-cellular calcium. Given that calcium can inactive complex III and complex I derived ROS, MitoTracker and mitoSOX co-stain were performed. As shown in Figure 4F, mitochondria derived ROS was increased as Cav1.3 was knocked out. After administration of Retenone, a complex I inhibitor, dose-dependent ROS reduction was observed in hair cells whose Cav1.3 was knocked out, but not in WT hair cells. Further, Antimycin A, a complex III inhibitor couldn’t affect Cav1.3 knock out induced ROS up-regulation (Figure 4G). These data suggested that Cav1.3 knock down induced ROS up-regulation was induced by reduced inactivation of complex I derived ROS. Taken together, Cav1.3 knock out decreased intra cellular calcium and subsequently resulted in reduction of complex I derived ROS inactivation.

### Cav1.3 knock down aggravated missing of hair cells in vivo after senescence induction and resulted in hearing impairment

**Figure 5:**
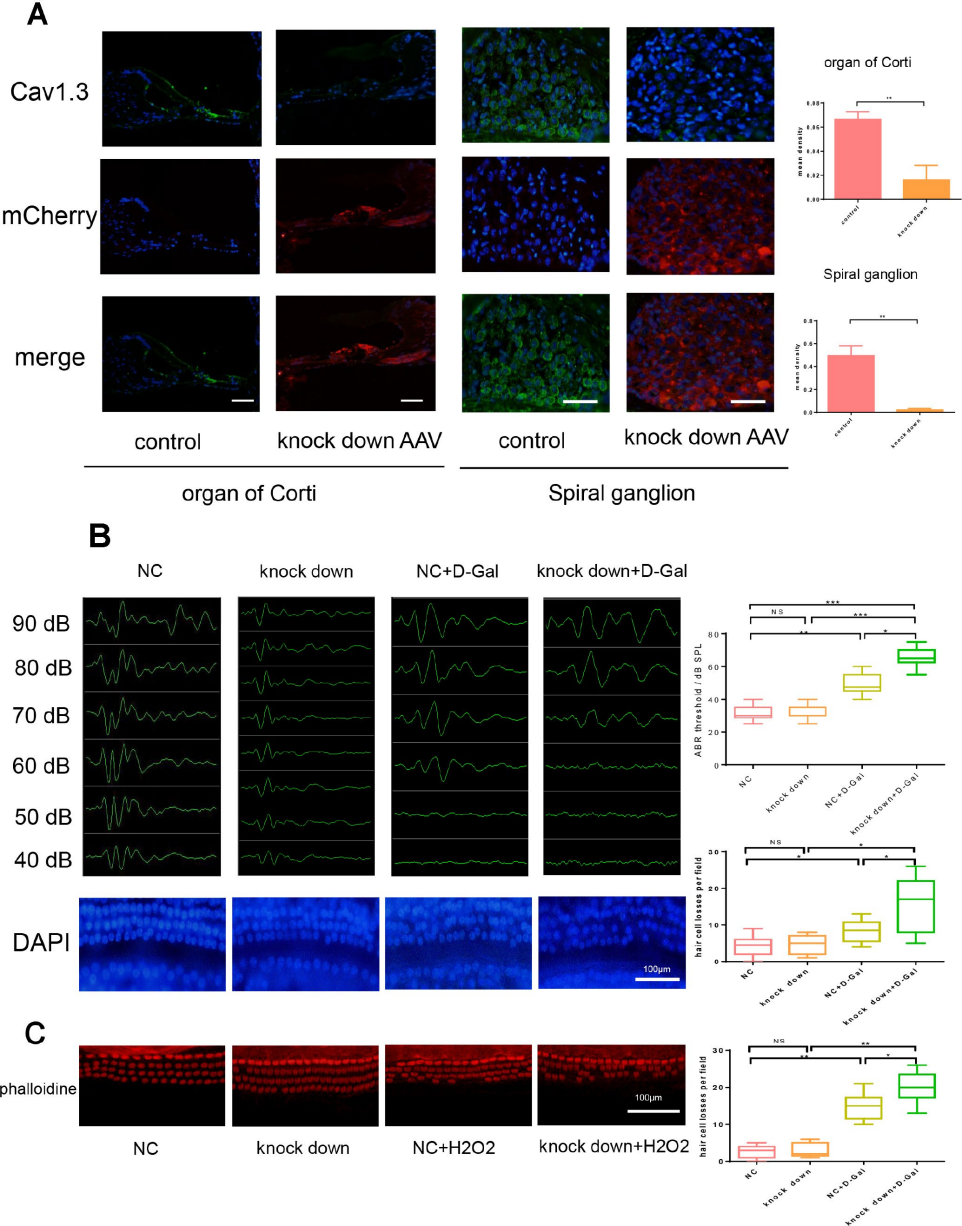
**Cav1.3 knock down aggravated the loss of hair cells after senescence induction and resulted in hearing impairment.** (**A**) immunofluorescence of CaV1.3 (green) and mCherry (red) in the organ of Corti (left) and spiral ganglion (right) of control and CaV1.3 knock down AAV group, nuclei was visualized by DAPI (magnification, ×400, scale bar: 50μm), the right panels are the quantitative analysis of CaV1.3 expression in organ of corti (top) and spiral ganglion (bottom). (**B**) auditory brainstem response (ABR) (top) and the whole cochlear basilar membrane after DAPI staining (bottom) of NC, CaV1.3 knock down, NC+D-Gal and CaV1.3 knock down+D-Gal group (n=6). (**C**) phalloidine staining for control and negative control AAV or Cav1.3 knock down AAV infected OC segment explants with H_2_O_2_ treatment (n=3), the right panel is the quantitative analysis. Error bars represent mean ± s.d.; *P<0.05; **P < 0.01; ***P < 0.001; ****P < 0.0001; n.s. not significant; by one-way analysis of variance (ANOVA) (**B**, **C**).

To evaluate role of Cav1.3 in vivo, Cav1.3 was knocked down and control adenovirus particle was administered via transbullar injection, the effect of knock down of Cav1.3 was assessed by western-blot of cochlea (Figure 5A). After 1 week, adenovirus infected mice were randomly divided into four group, one of Cav1.3 knock down group and control virus infection group was subjected to D-Gal injection to induce senescence. After 6 weeks, auditory brainstem response (ABRs) was evaluated, as shown in Figure 5B, the characteristic ABR waveforms were decreased after aging induction, and Cav1.3 knock down aggravated this effect, no significant changes in ABR sensitivity was observed after Cav1.3 was knocked down. Cochlea basilar membrane stretched preparation was used to study underlying mechanism. As shown in Figure 5B, slight hair cell losses were observed after induction of aging, and the hair cell loss was increased after Cav1.3 knock-down, which suggested that down-regulation of Cav1.3 can aggravate age-related hair cell loss, again, Cav1.3 knock down itself do not result in losses of hair cells.

To further confirm the role of Cav1.3 on the cochlear hair cells, the organ of Corti (OC) of newborn mice (P3-P5) was isolated and subjected to 3D culture. After induction of Cav1.3 knock down AAV or control AAV, the organ was treated with 0.5Μm hydrogen peroxide for 1 hour and cultured in hydrogen peroxide-free medium for another 24 hours. As shown in Figure 5C, similar with the results of in vivo studies, Cav1.3 knock-down enhanced hydrogen peroxide induced hair cell losses.

Taken together, Cav1.3 knock down aggravated the loss of hair cells after induction of aging and resulted in impairment of hearing.

## DISCUSSION

Presbycusis is a common term used for age-related hearing loss. This term including all conditions that lead to hearing loss in elderly people. The clinical presentation of ARHL is characterized by reduced hearing sensitivity and speech understanding, and this disorder is classified into- sensory, strial, and neural [22]. The most common causes for ARHL is damage, loss and death of sensory hearing cells in the cochlea [23]. Several molecular changes have been proposed for ARHL, such as reduction in oxygen delivery, genetic mutation and a significant increase in the production of reactive oxygen species (ROS) [14, 24, 25]. ROS is mainly generated in the mitochondria [26, 27], and this can damage key mitochondria components, such as mitochondrial DNA (mtDNA), mitochondrial membranes, and nuclear DNA that affect function of mitochondria. The impairment of mitochondrial function increase production of ROS, this creates “vicious cycle” which can finally trigger the cascade of apoptosis and cell death [24, 28–30].

The C57BL/6 strain has been most extensively studied as a model of early-onset hearing loss, Our previously research was based on C57 early-onset hearing loss model and found expression of Cav1.3 was changes during the process of aging, Cav1.3 is mainly expressed in CNS, it has been reported that Ca^2+^ dysfunction plays an important role in age-related neurodegeneration [31]. Chen et al [13] reported that Cav1.3 expressed in cochlea gradually reduced in the aging mice and the down regulation of Cav1.3 might be associated with ARHL, however the underlying mechanism remain unknown. In this study, expression of Cav1.3 was confirmed by IF, which showed that Cav1.3 was predominantly expressed in inner hair cells (IHCs), outer hair cells (OHCs), and spiral ganglion (SG) neurons. The expression was significantly down-regulated in aging mice, and this result was in agreement with previous reports [13]. Age-related Cav1.3 expression in other part of auditory pathway, including inferior colliculus, cochlear nucleus and auditory cortex were also investigated.

The function of Cav1.3 down regulation was investigated in vitro, and it was found that the hair cells were vulnerable to ROS stresses. Down-regulation of Cav1.3 decreased Ca^2+^ influx through to Cav1.3 channel and subsequently resulted in hyperpolarize of resting membrane potential [32]. Down-regulation of Cav1.3 also resulted in increased generation of ROS from mitochondria and after administration of Ionmycin or complex I inhibitor, the effect was partly impaired which indicated that down regulation of Cav1.3 resulted in reduction of intra cellular calcium and subsequently reduced inactivation of ROS generated from complex I of mitochondria, and finally triggered cell apoptosis under ROS stresses.

The effect was further confirmed in vivo, after Cav1.3 was knocked down by injection of AAV, the mice were more vulnerable to aging as they exhibited higher hearing threshold and increased loss of hair cells. Since knock down of Cav1.3 can interfere with the functioning of stria vascularis which can also result in losses of hair cells, 3D organ culture was used to further confirm the proposed theory, and it was found that hair cell in the organ of Corti was more vulnerable to ROS stresses as Cav1.3 was knocked down, the result was in agreement with in vitro experiment.

But our work still has some limitation. First, in this study, only male C57 was used to investigate function of Cav1.3, expression of Cav1.3 might be different between male and female animal as changes of hearing sensitivity during aging processes is different between male and female animal [33]. Second, our work mainly focuses on relationship between expression of Cav1.3 and apoptosis of hair cells, so only total expression of Cav1.3 in cochlea was evaluated rather than record them along tonotopic map.

In summary, these results indicate that down regulation of Cav1.3 expression in cochlea promotes age-related hearing losses through augment of ROS from complex I of mitochondria in hair cells and provides a possible target for prevention of ARHL.

## MATERIALS AND METHODS

### Ethic statement

The care and experimental treatment of the animals were approved by the Animal Research Committee of Tongji Medical College of Huazhong University of Science and Technology.

### Animals

C57BL/6J male mice were purchased from the Model Animal Research Center of Tongji Medical College of Huazhong University of Science and Technology (Wuhan, China). Mice were randomly divided into five groups: group A (4 weeks), B (8 weeks), C (16 weeks), D (32 weeks), E (48 weeks), each group consisted of 5 mice. The care and experimental treatment of the animals were approved by the Animal Research Committee of Tongji Medical College of Huazhong University of Science and Technology.

### Tissue preparation

All the deeply anesthetized animals were decapitated, the brains were removed immediately and fixed by immersing in 4% paraformaldehyde in 0.1 mM phosphate-buffered saline (PBS), pH 7.4, overnight at 4°C. The brains were embedded in O.C.T. compound (Sakura, USA) the following day. Before use, the brains were cryosectioned at 6 μm thickness for immunofluorescence, mounted on glass slides and stored at −20°C.

The auditory cortex was identified in accordance with the mouse atlas of Paxinos and Watson (2001). Tissues within the anterior and posterior ectosylvian sulci (roughly delineating AI) were dissected. For RNA preparations, auditory cortex tissues were dissected with small forceps and immediately frozen in liquid nitrogen and stored at -80°C before use.

### Immunofluorescence staining

The cochlea and Auditory Cortex sections were thawed and dried for 30 min at room temperature, permeabilized with 0.1% Triton X-100(Sigma) in 50mM PBS for 20 min, washed 3 times and blocked with 10% goat serum for 1 h at room temperature. After overnight incubation with the primary antibody, rabbit anti-CaV1.3 calcium channel polyclonal antibody (1:50; Alomone labs, Israel) or mouse anti-Myo7a antibody (1:100; Santa Cruz, CA) at 4°C the sections were washed three times with PBS and incubated using Dylight 488 conjugated goat anti-rabbit IgG or Dylight 594 conjugated goat anti-mouse (1:500; Multi-Sciences, Hangzhou, China) for 1 h at room temperature. The sections were then washed with PBS and costained with 10μg/ml DAPI (Sigma, USA) for 20 min. After finally washing with PBS, the slides were covered with glass cover slips and observed under an Olympus Fluoview 500 IX 71 confocal microscope (Olympus, Tokyo, Japan). The images were digitally recorded at the same magnification and time of exposure. For hair cells examination, the surface of the OC was prepared by removing the otic capsule and tectorial membrane of the fixed cochlea. The dissected specimens were labeled with anti-CaV1.3 and DAPI before observation.

### Quantitative real-time polymerase chain reaction (q-PCR)

The Auditory Cortex(AC), inferior colliculus and cochlear nucleus tissues were dissected and total mRNA was extracted using TRIzol Reagent (Invitrogen, Carlsbad, California, USA) following the manufacturer’s protocol. The extracted mRNA (1μg) was reverse transcribed into cDNA using ReverTra-Plus-TM (Toyobo, Osaka, Japan). Real-time PCR was performed on a LightCycler System 2.0 (Roche, Mannheim, Germany) using SYBR Premix EX Taq kit (Takara, Dalian, China). The primer sequences of CaV1.3 (CACNA1D) were: 5’-TGC ACA GAT GAA GCC AAA AG-3’ for forward and 5’-ACA GAA CCA ACG TTC TCA CC-3’ for reverse. A housekeeping gene, β-actin (forward:5’-GCG CAA GTA CTC TGT GTG GA-3’, reverse: 5’-GAA AGG GTG TAA AAC GCA GC-3’), was used as an internal control. PCR was performed at 95°C for 5 min, then 95°C (45 s), 56°C (30 s), and 72°C (45 s), followed by a 10 min extension at 72°C for 40 cycles. Each sample was run in triplicate and averaged. The relative gene expression was calculated by 2^-△△Ct^ method.

### Cell culture, reagents, transfections

The hair cell line HEI-OC1 cells, obtained from Sun Yat-sen Memorial Hospital, were incubated in Dulbecco's Eagle's medium (DMEM; Gibco BRL, Gaithersburg, Md., USA) containing 10% fetal bovine serum (FBS; Gibco BRL, USA) without antibiotics at 33°C under 10% CO_2_ (permissive conditions). For senescence induction, cells were treated with 5μM hydrogen peroxide (H_2_O_2_; Sigma, USA) for 1 h before cultured in fresh growth medium for 24h, or 20mg/ml D-galactose for 48h. Ionmycin (Medchem express, USA), a Calcium ionophore; Rotenone (Medchem express, USA), a complex I inhibitor; Antimycin A (Abcam, UK), a complex III inhibitor. CaV1.3 knock out and control lentiviral particle, CaV1.3 knock down and control Adenovirus and Adeno-association virus (AAV) were purchased from Genechem Co (China, shanghai). For lentiviral transduction, 5000 cells/well were seeded in 96 well tissue culture plates and infected the following day with lentiviral particles at a MOI of 10 in the presence of 10 mg/ml polybrene, purchased from Santa-Cruz Biotechnology (Dallas, TX). After infection, HEI-OC1 was selected with 5μg/ml puromycin, purchased from Life Technologies (Carlsbad, CA).

### Flow cytometry staining and analysis

The Auditory Cortex (AC) tissues of different ages were dissected, then subjected to 2 mg/ml Papain (Sigma, USA) and DNase digestion for 30min at 37°C. After digestion, it was passed through a 70 μm mesh (BD Falcon, CA, USA). We centrifuged the filtrate and washed the remaining cells with PBS. The cells for analysis were re-suspended in FACS staining buffer for flow cytometry. Cells were stained with anti-CaV1.3 (1:50) for 1 h, and washed 3 times with PBS, then stained with anti-rabbit, APC (1:1000; Cell Signaling Technology, USA) and anti-NeuN, FITC (1:100; Cell Signaling Technology, USA) for 30min at 37°C. Labeled cells were analyzed on BD FACSVerse Flow Cytometer, and the data were processed using FLOWJO software (Treestar).

### Western blot

The tissues of Auditory Cortex were separately dissected and pooled to obtain equalized protein content (30 μg). Following the standard Western blot technique, the proteins were respectively electrophoresed through a 10% SDS-PAGE and transferred to immobilon polyvinylidene difluoride membrane (Millipore, Billerica, Massachusetts, USA). The membranes were blocked for 1 h at room temperature in 5% non-fat milk/TBS (10 mM Tris-HCl (pH8.0), 150 mM NaCl, 0.05% tween 20). Proteins were probed with primary rabbit anti-CaV1.3 antibody (1:200; Alomone labs, Israel) overnight at 4°C and incubated with GAPDH antibody (1:2000; Sigma, USA) as the internal control. After rinsing three times (10 min each) with TBS, the membrane was incubated with horseradish peroxidase-conjugated secondary antibody against rabbit IgG (1:5000, Amersham Bioscience, Piscataway, NJ) for 1 h at room temperature. After washout, the membrane was developed using ECL reagents (Pierce, Rockford, Illinois, USA) and visualized using a chemiluminescence system (PTC-200; Bio-RAD Laboratories, Hercules, California, USA). All western blots were repeated three times.

### Patch clamp

Current was recorded by EPC10 patch-clamp amplifiers (HEKA electronic, Lambrecht, Pfalz, Germany), 2 kHz filtering, digitized to 10 kHz, and then stored in the computer. The non-linear capacitance (NLC) and membrane potential (MP) of HEI-OC1 cells (n=5) were recorded in the voltage clamp mode using whole-cell patch clamp technique. External solutions of NLC were recorded (mM): 100 NaCl, 20 CsCl, 20 TEACl, 2 CaCl_2_, 1.47 MgCl_2_, 2 CoCl_2_, and 10 HEPES. PH was adjusted to 7.3 with NaOH. Internal solution containing (mM): 140 CsCl, 2 MgCl_2_, 10 EGTA, and 10 HEPES, PH adjusted to 7.2 using CsOH. Boltzmann equation was used to fit NLC. The external solutions for recording MP included (mM): 142 NaCl, 5 KCl, 1.5 CaCl_2_, 2 MgCl_2_, 10 HEPES, 5.6 glucose. The internal solution contained (mM): 148 KCl, 0.5 CaCl_2_, 2 MgCl_2_, 10 HEPES, and 1 EGTA. PH was adjusted to 7.4 with KOH. The impedance of the glass electrode was 5-8 m in whole cell recording. Data analysis was carried out with fitmaster software, and origin 8.0 was used for drawing.

### 3D organ culture

Newborn C57BL/6J mice (P3-P5) were used (n=3). After the temporal bone was removed from the skull, the organ of Corti (OC) explant was isolated in cold, sterile, buffered saline glucose solution. The OC explant was cut into 3 segments corresponding to basal, middle and apical turns. Each segment was cultured onto Corning Matrigel Basement Membrane Matrix (BD Biocoat, USA). The OC explant isolated from another ear in the same mouse was served as self-control. The data-processing was based on the treated group and the self-control group from the same animal. The OC explants were infected with CaV1.3 knock down or control Adenovirus and cultured at 37°C in an incubator with 5% CO_2_ for 24 h, the OC explants were treated by 0.5 μM H_2_O_2_ for 1 h, then cultured in fresh growth medium for 24h. The OC segment explants were fixed in 4% paraformaldehyde at room temperature for 30 min and then permeabilized with 0.2% Triton X-100 in PBS for 30 min. For hair cell labeling, the epithelia were stained with tetramethyl rhodamine isothiocyanate (TRITC)-conjugated phalloidin (5μg/ml; Sigma–Aldrich, Saint Louis, USA) and examined under a fluorescence microscope. The hair cells were counted over a longitudinal distance of 100μm in three separated microscopic fields for each segment. Cells were considered missing when there was a gap in the normal arrays. A mean value was calculated for each specimen and at least 6 explants were used for each group.

### Animal experiments

The middle ears of C57BL/J male mice were inoculated via transbullar injection method with 5 μl Cav1.3 knock down Adeno-associated virus (AAV) labeled with mCherry, and the negative control (NC) group was inoculated equivalent control AAV. After 1 week, the 0.25 ml/10g 5% D-galactose were injected into mice for 6 weeks to induce senescence. The distribution and intensity of red fluorescence were estimated by fluorescent microscope and the ABR threshold was recorded.

### Auditory brainstem response recording

Auditory brainstem response (ABR) was recorded by a Nicolet Compass ABR System (Natus Medical Inc., San Carlos, California, USA). Mice were anesthetized with an intraperitoneal injection of tribromoethanol at 300 mg/kg. ABR was evoked by click stimulation and averaged by 1000 times. The stimulus level was started at 90 dB SPL and descended in a 10 dB SPL step until a visually discernible ABR waveform was undetectable.

### Statistical analyses

All the data were presented as the mean±SEM. One-way analysis of variance (ANOVA) was used to analyze the differences among groups using SPSS 13.0 (SPSS Inc., Chicago, Illinois, USA). Pair-wise comparisons were also carried out between the groups using the Student–Newman–Kuels (SNK) test. A P value less than 0.05 was considered to be statistically significant.
